# Supplementary material for: Anti-Cancer Activity and Phenolic Content of Extracts Derived from Cypriot Carob (Ceratonia siliqua L.) Pods Using Different Solvents
Source: Molecules. 2021 Aug 19;26(16):5017. doi: 10.3390/molecules26165017 (PMC8401790; doi:10.3390/molecules26165017)
Supplement: Supplementary file 1 [file molecules-26-05017-s001.zip › molecules-1304681-supplementary.pdf]

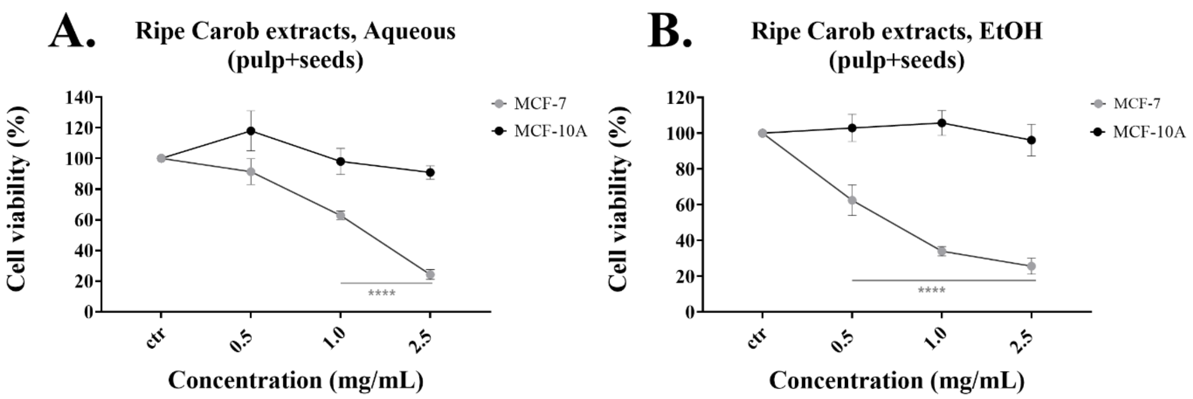

Figure S1. Antiproliferative effect of extracts produced from the whole ripe carob fruit extracted with (A) Water and (B) EtOH, on MCF-7 and MCF-10A cell lines. Treatment was performed for 48 h. *p* values: * < 0.05, ** < 0.01, *** < 0.001, **** < 0.0001 compared to control.

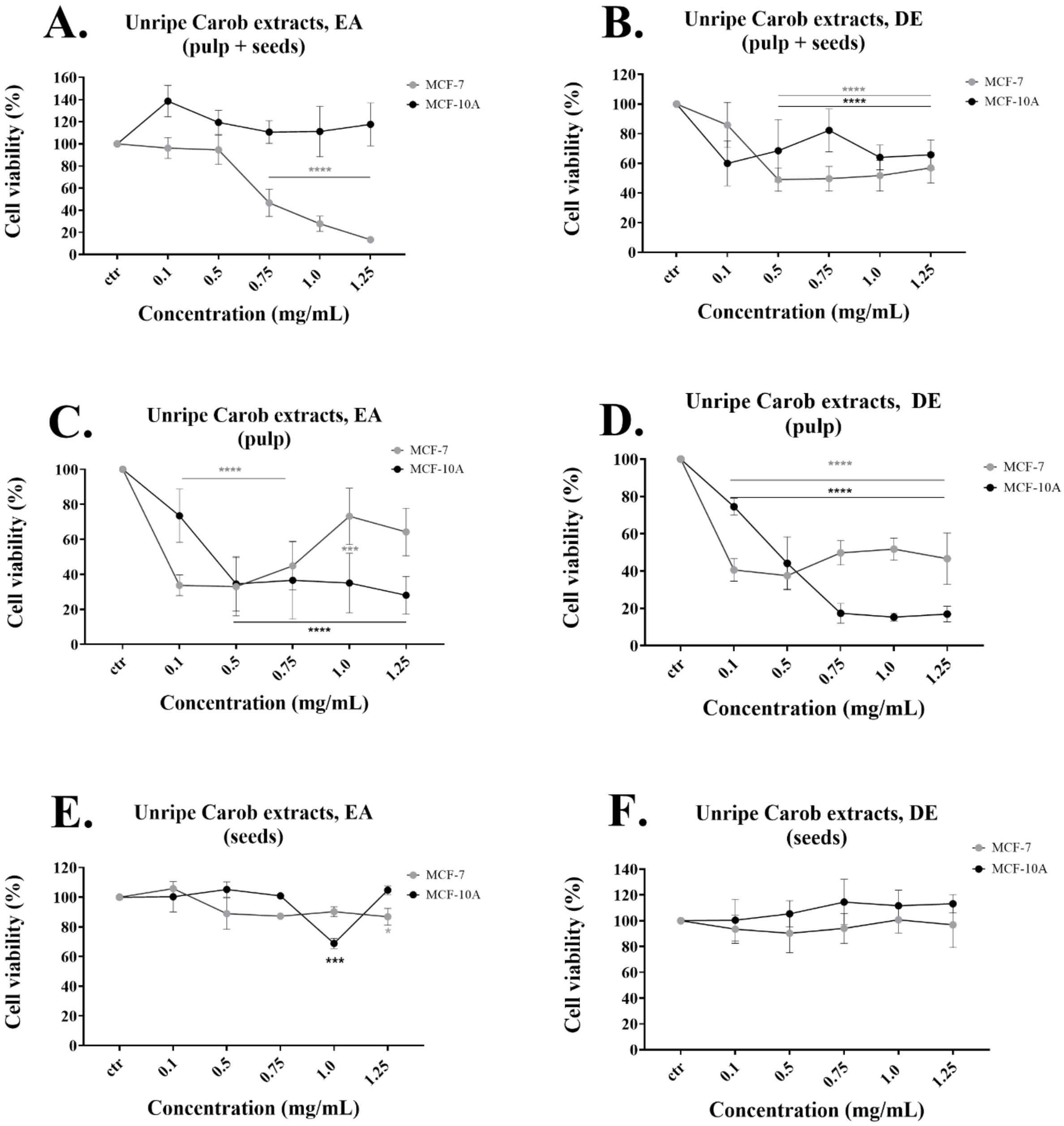

Figure S2. Comparison of the effects on cell viability using unripe carob extracts. Cells were treated for 48 h with extracts produced from the whole unripe carob using (A) EA and (B) DE, from the unripe pulp using (C) EA and (D) DE and from the unripe seeds using (E) EA and (F) DE. *p* values: * < 0.05, ** < 0.01, *** < 0.001, **** < 0.0001 compared to control.

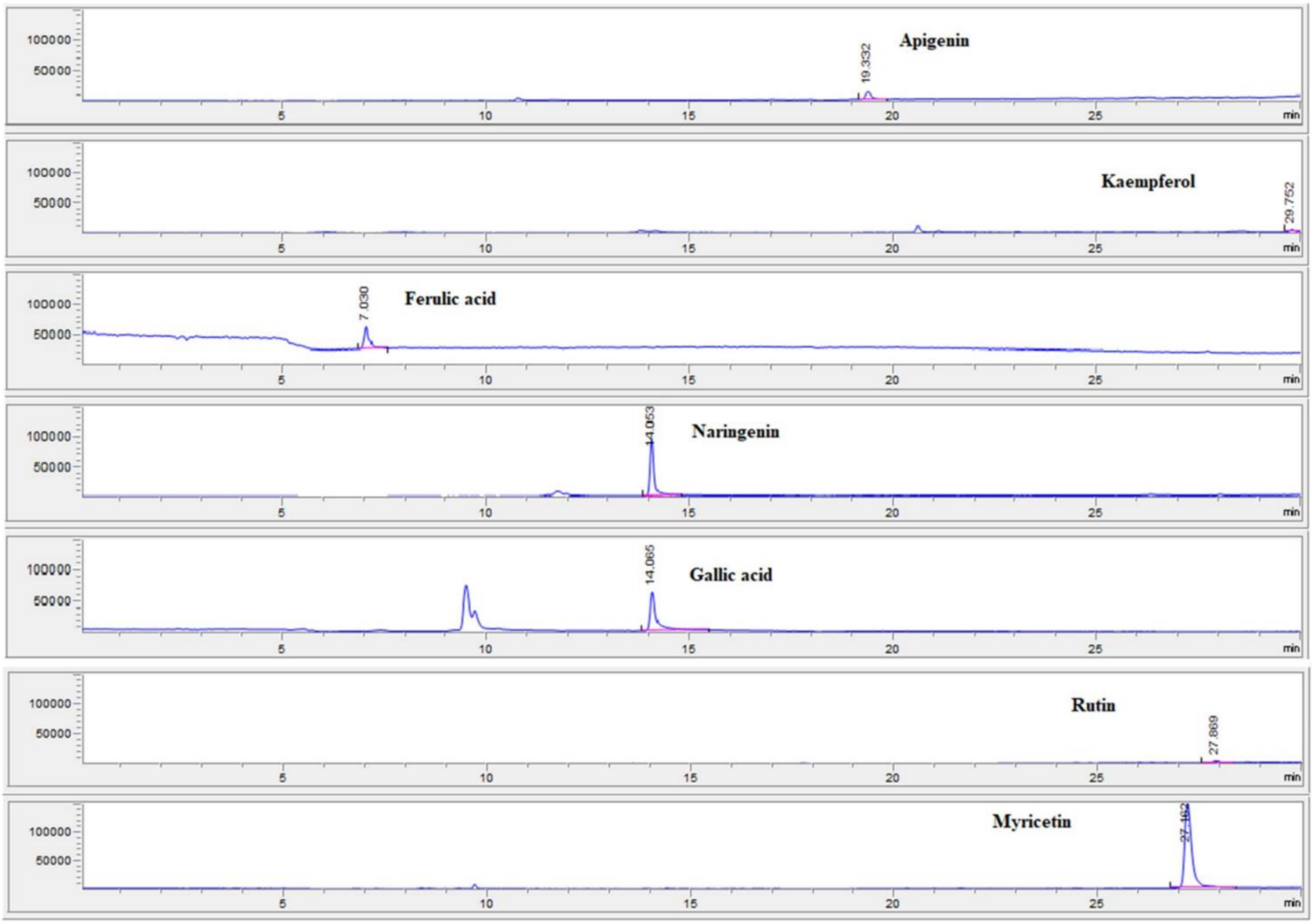

Figure S3. Chromatograms of the 250-ppb EA ripe pulp + seeds carob extract in SIM mode.

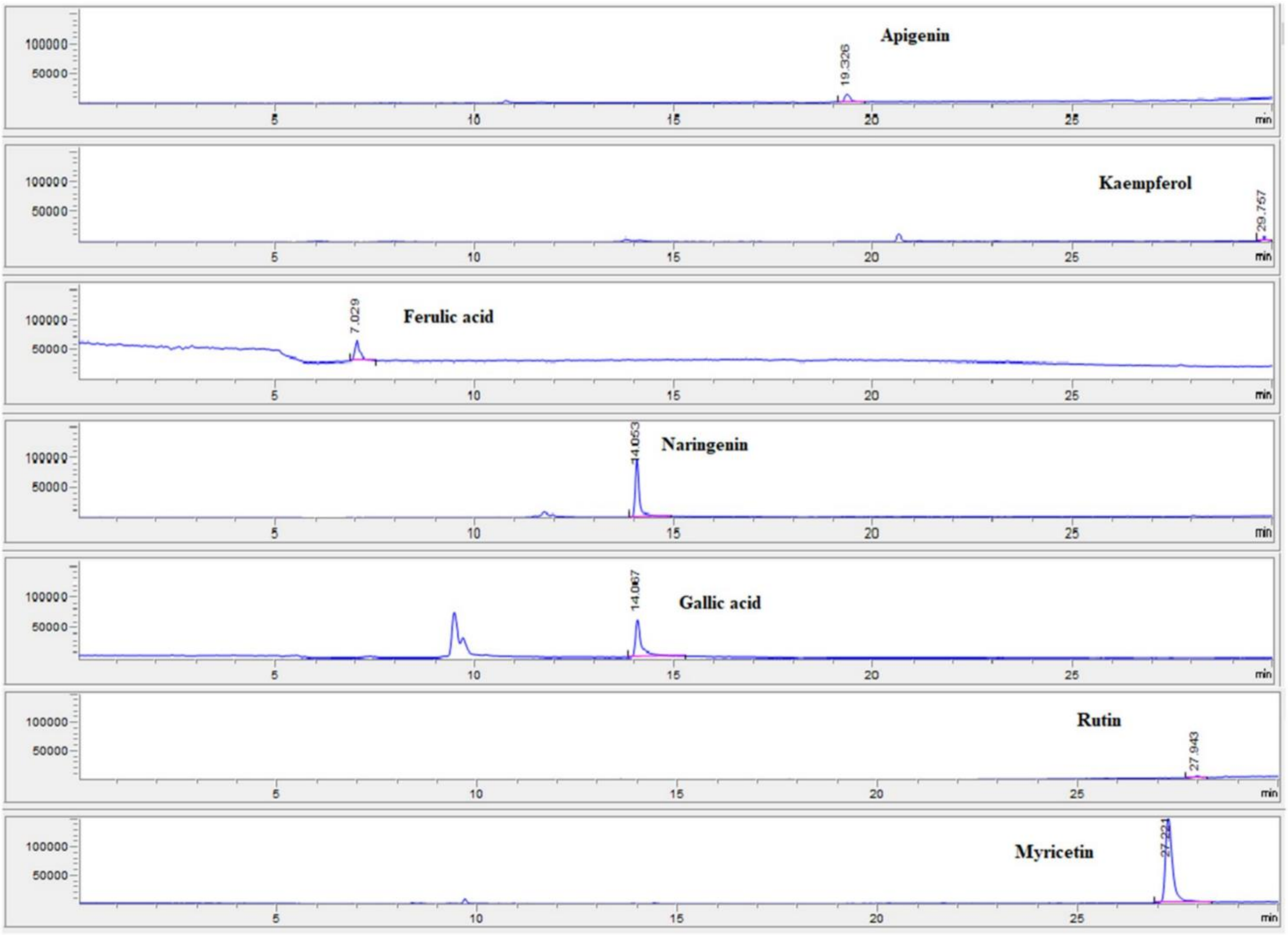

Figure S4. Chromatographs of the 250-ppb DE ripe pulp + seeds carob extract in SIM mode.

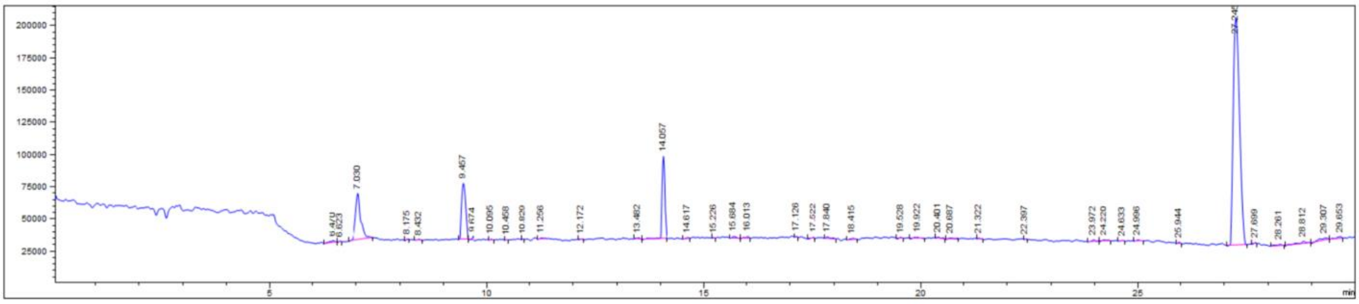

Figure S5. Total ion chromatogram of DE extract.

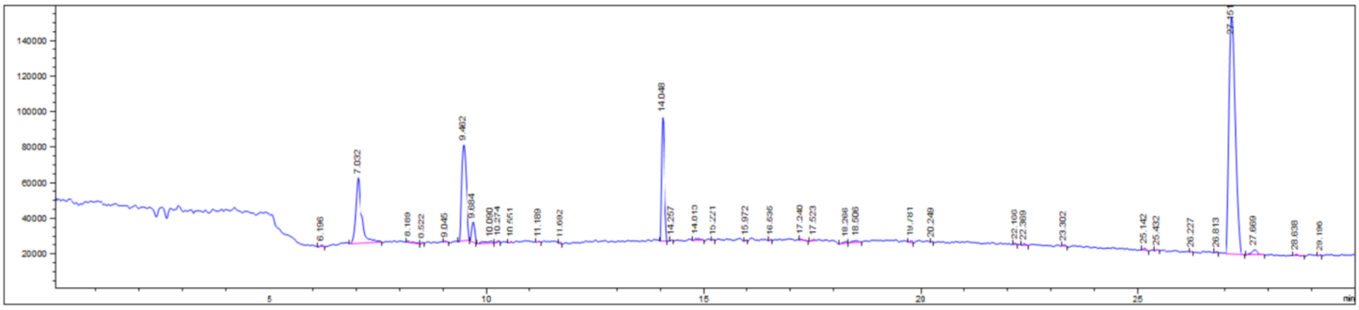

Figure S6. Total ion chromatogram of EA extract.

Table S1. Retention times, *m/z* of each compound, regression equations, coefficients of determination (R^2), LODs and LOQs (N = 3)*, and % relative standard deviation (%RSD) of the peak areas (N = 5)**.

Compound	$[M + H]^+$	$t_R \pm SD$	Regression equation	R^2	LOD ng/mL	LOQ ng/mL	RSD% EA	RSD% DE
**Apigenin**	271.052	19.33 ± 0.01	y = 25276 x + 76662	0.9993	0.486	1.473	1.12	2.06
**Myricetin**	319.235	27.17 ± 0.02	y = 17673 x + 63571	0.9992	2.741	8.309	0.37	0.35
**Rutin**	610.517	27.88 ± 0.03	y = 2908.5 x + 14265	0.9983	1.018	3.085	1.19	0.47
**Naringenin**	273.257	14.05 ± 0.00	y = 17701 x − 156115	0.9978	2.702	8.187	0.80	0.46
**Ferulic acid**	195.180	7.03 ± 0.00	y = 25111 x + 309986	0.9994	0.367	1.111	1.31	-
**Kaempferol**	287.230	29.75 ± 0.00	y = 4114.1 x + 4671.7	0.9953	1.700	5.153	1.68	0.74
**Gallic acid**	171.120	14.06 ± 0.00	y = 219786 x − 108299	0.9995	0.369	1.117	0.42	0.02

* Three replicate runs were performed. ** Five replicate runs were performed.
